# Supplementary material for: The diverse functions of viral microproteins
Source: bioRxiv. 2026 Aug 24:2026.08.23.746553. Preprint. [Version 1] doi: 10.64898/2026.08.23.746553 (PMC13622983; doi:10.64898/2026.08.23.746553)
Supplement: Supplementary file 1 [file NIHPP2026.08.23.746553V1-supplement-1.pdf]

bioRxiv preprint doi: https://doi.org/10.64898/2026.08.23.746553; this version posted August 24, 2026. The copyright holder for this preprint (which was not certified by peer review) is the author/funder, who has granted bioRxiv a license to display the preprint in perpetuity. It is made available under a CC-BY 4.0 International license.

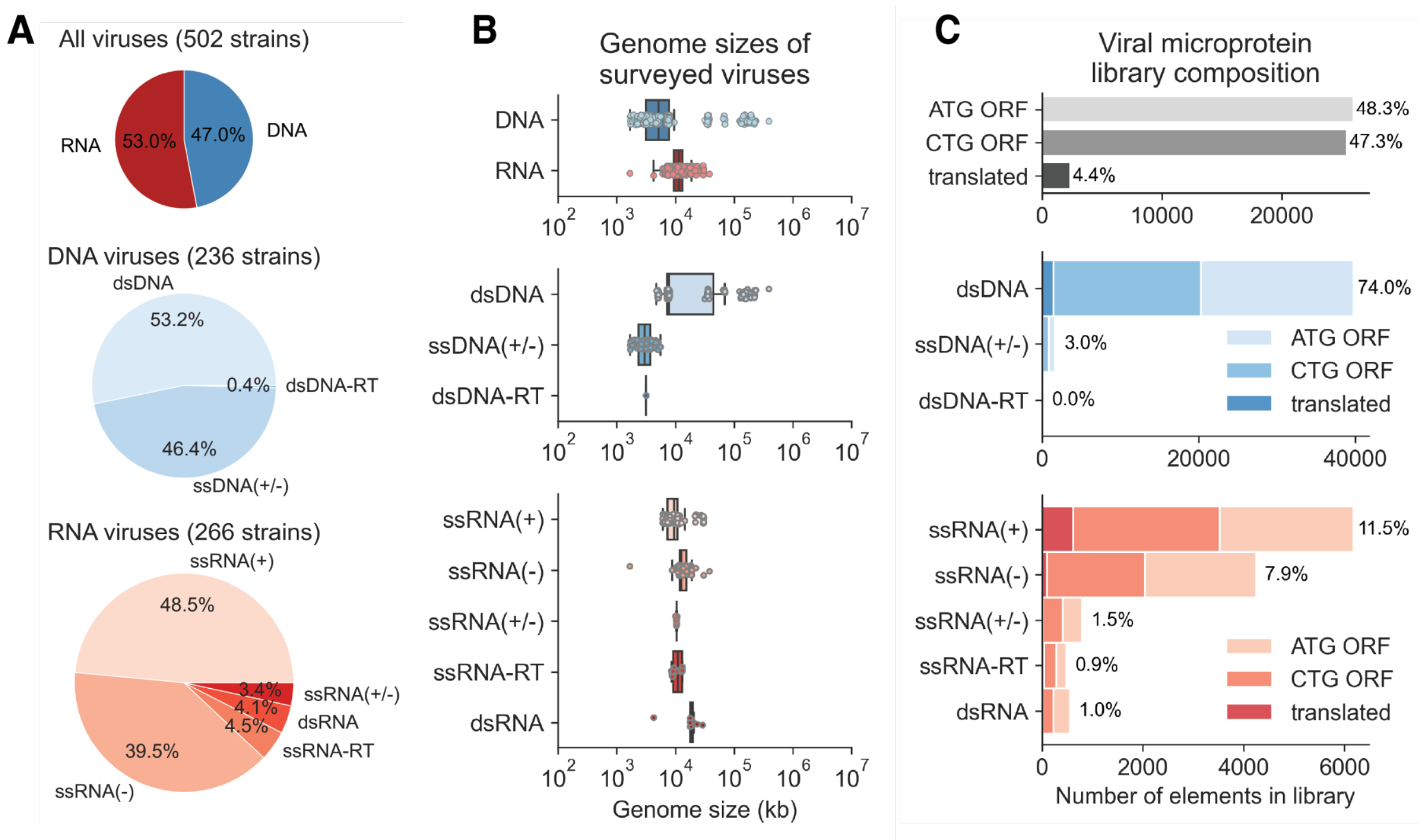

Fig. S1. Survey of viral genomes for smORFeome curation. (A) Baltimore classifications of the viral genomes. (**B**) Genome sizes. (**C**) Fraction of ORFs derived from each classification.

5

bioRxiv preprint doi: https://doi.org/10.64898/2026.08.23.746553; this version posted August 24, 2026. The copyright holder for this preprint (which was not certified by peer review) is the author/funder, who has granted bioRxiv a license to display the preprint in perpetuity. It is made available under a CC-BY 4.0 International license.

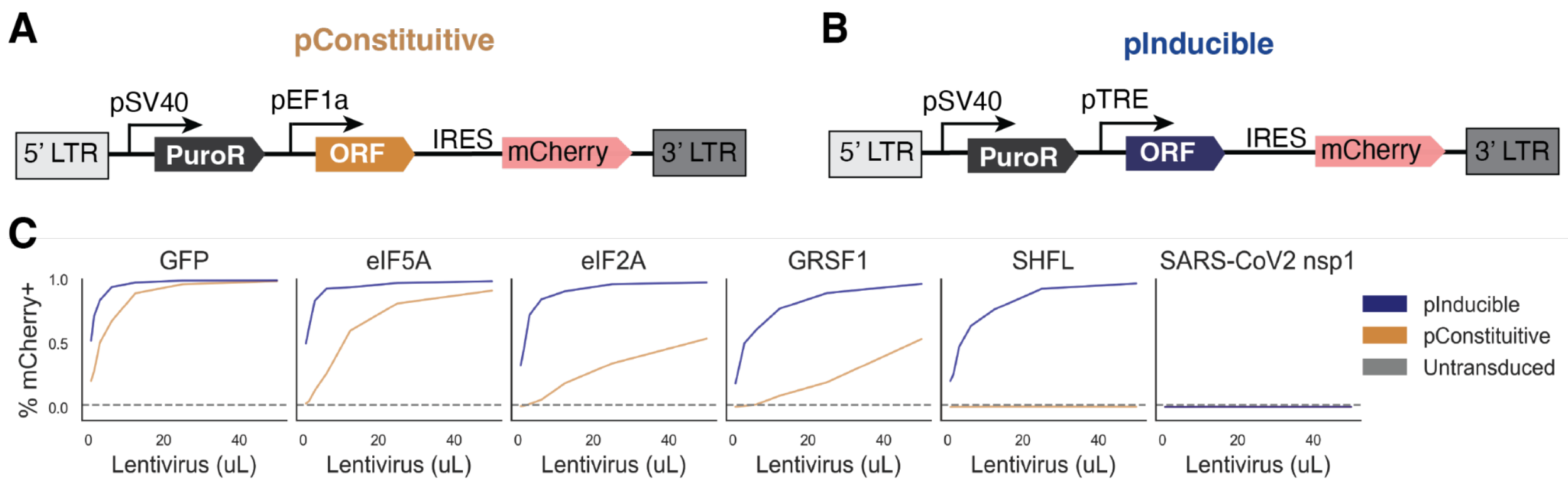

Fig. S2. Toxic ORFs do not produce viable lentivirus. Lentiviral vector designs for (**A**) constitutive and (**B**) inducible ORF expression. ORFs are encoded on a polycistronic transcript with an IRES-mCherry. (**C**) Fraction of mCherry-positive cells 4 days after transduction and 1 day of dox induction with indicated ORFs titrated with different volumes of lentivirus.

bioRxiv preprint doi: https://doi.org/10.64898/2026.08.23.746553; this version posted August 24, 2026. The copyright holder for this preprint (which was not certified by peer review) is the author/funder, who has granted bioRxiv a license to display the preprint in perpetuity. It is made available under a CC-BY 4.0 International license.

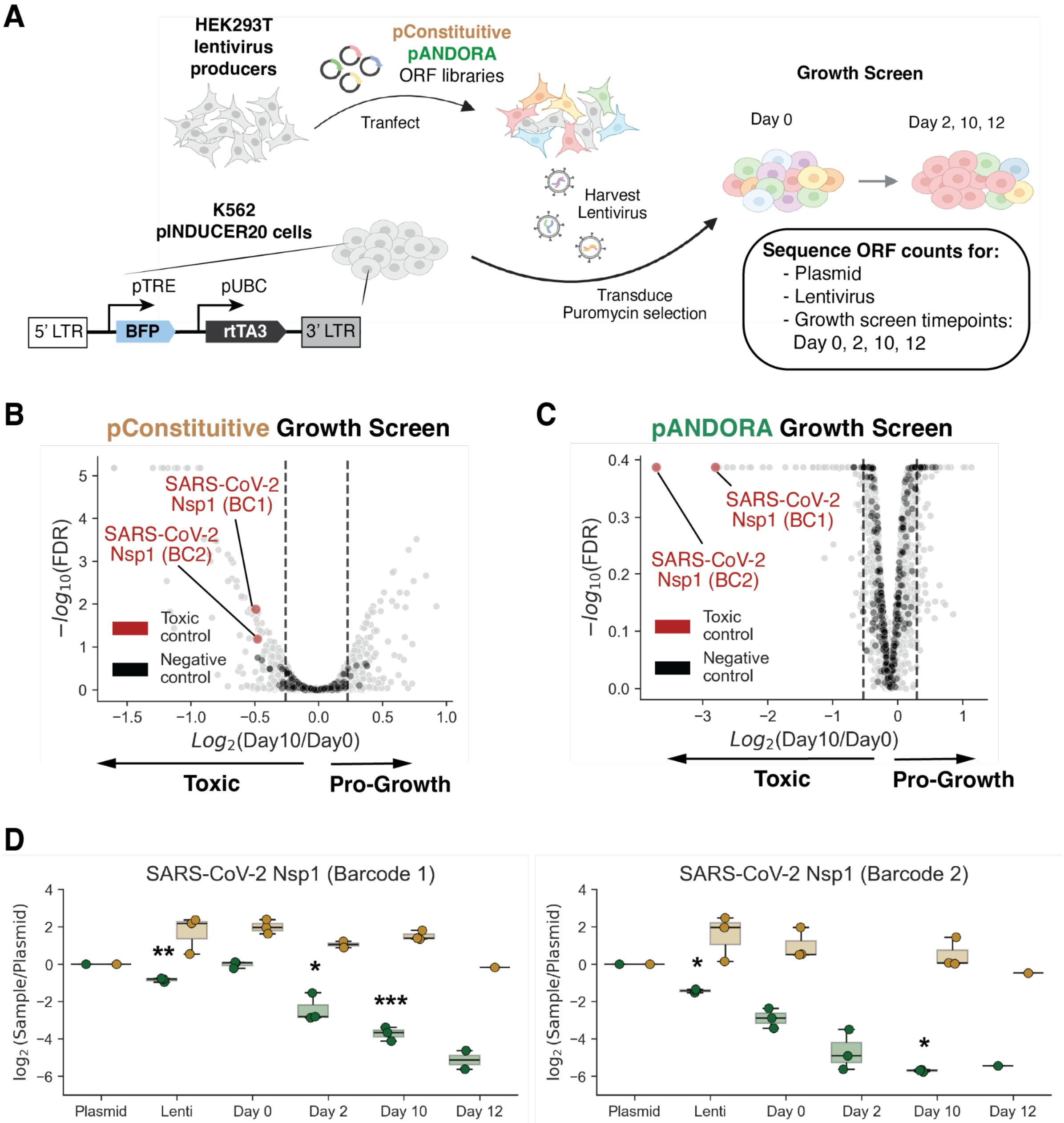

Fig. S3. Leakiness of inducible ORF vectors impacts sensitivity of toxic ORF detection in pooled ORF screens. (**A**) Schematic of the screen design for evaluating the sensitivity of the pConstitutive and pANDORA vectors cloned with the 66-88aa smORF library. K562 growth screen results for the (**B**) pConstitutive and (**C**) pANDORA libraries. (**D**) SARS-CoV-2 Nsp1 ORF counts at the plasmid, lentivirus, day 0, day 2, day 10, and day 12 stages.

bioRxiv preprint doi: https://doi.org/10.64898/2026.08.23.746553; this version posted August 24, 2026. The copyright holder for this preprint (which was not certified by peer review) is the author/funder, who has granted bioRxiv a license to display the preprint in perpetuity. It is made available under a CC-BY 4.0 International license.

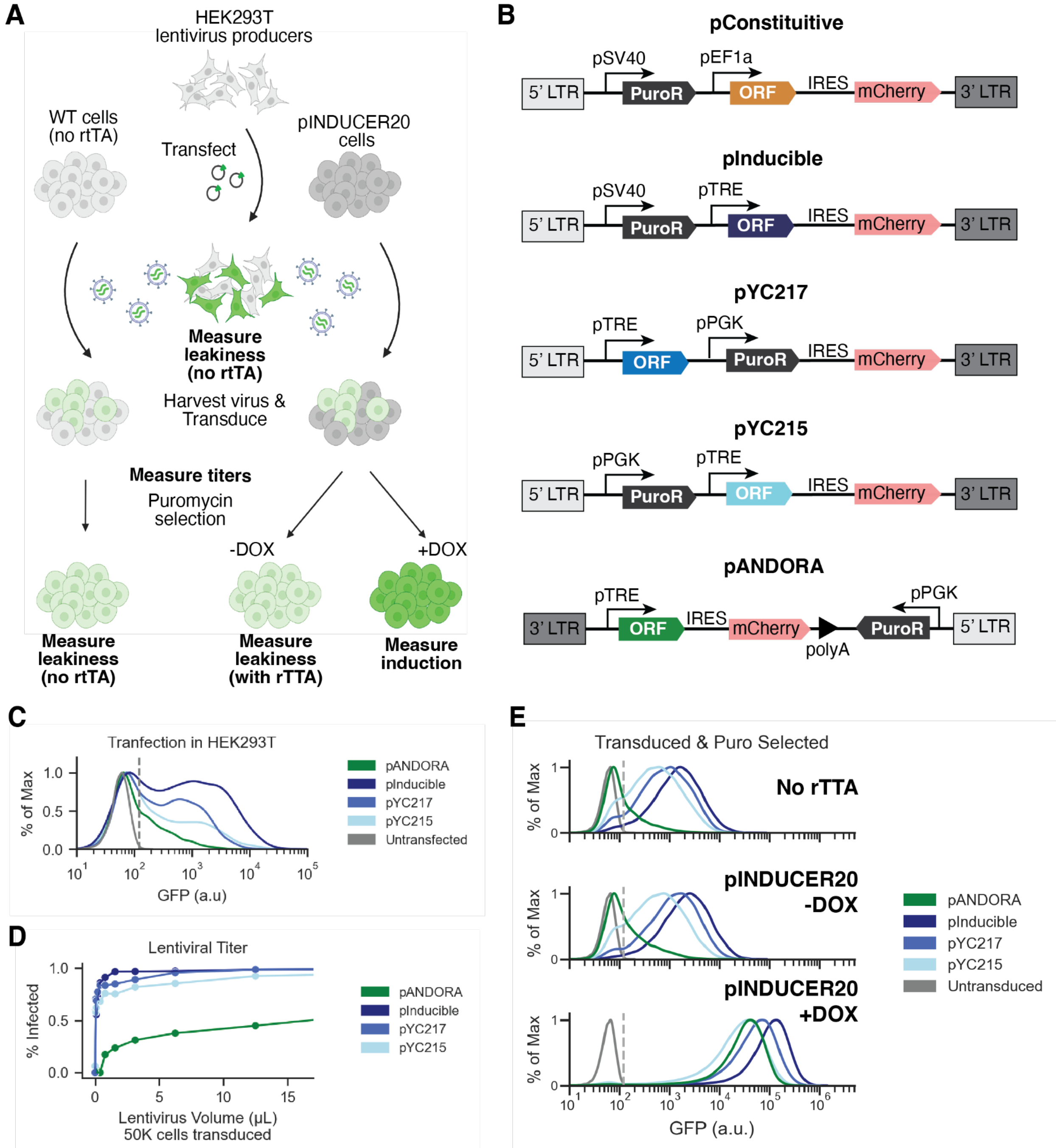

Fig. S4. Optimization of a minimally leaky inducible vector for toxic ORF expression. (**A**) Schematic for examining titers, induction, and leakiness at different stages of the genetic screening pipeline. (**B**) Vector designs for inducible ORF overexpression; GFP was cloned into all ORF vectors for testing. (**C**) HEK293T producer cells that do not express rtTA were transfected, and GFP expression was examined by flow cytometry 48 h after transfection. (**D**) K562 cells were transduced with the indicated volumes of lentivirus produced from GFP ORF vectors, grown in doxycycline media for 72 h, and assessed for the fraction of GFP-positive cells. (**E**) GFP levels 72 h after transduction.

bioRxiv preprint doi: https://doi.org/10.64898/2026.08.23.746553; this version posted August 24, 2026. The copyright holder for this preprint (which was not certified by peer review) is the author/funder, who has granted bioRxiv a license to display the preprint in perpetuity. It is made available under a CC-BY 4.0 International license.

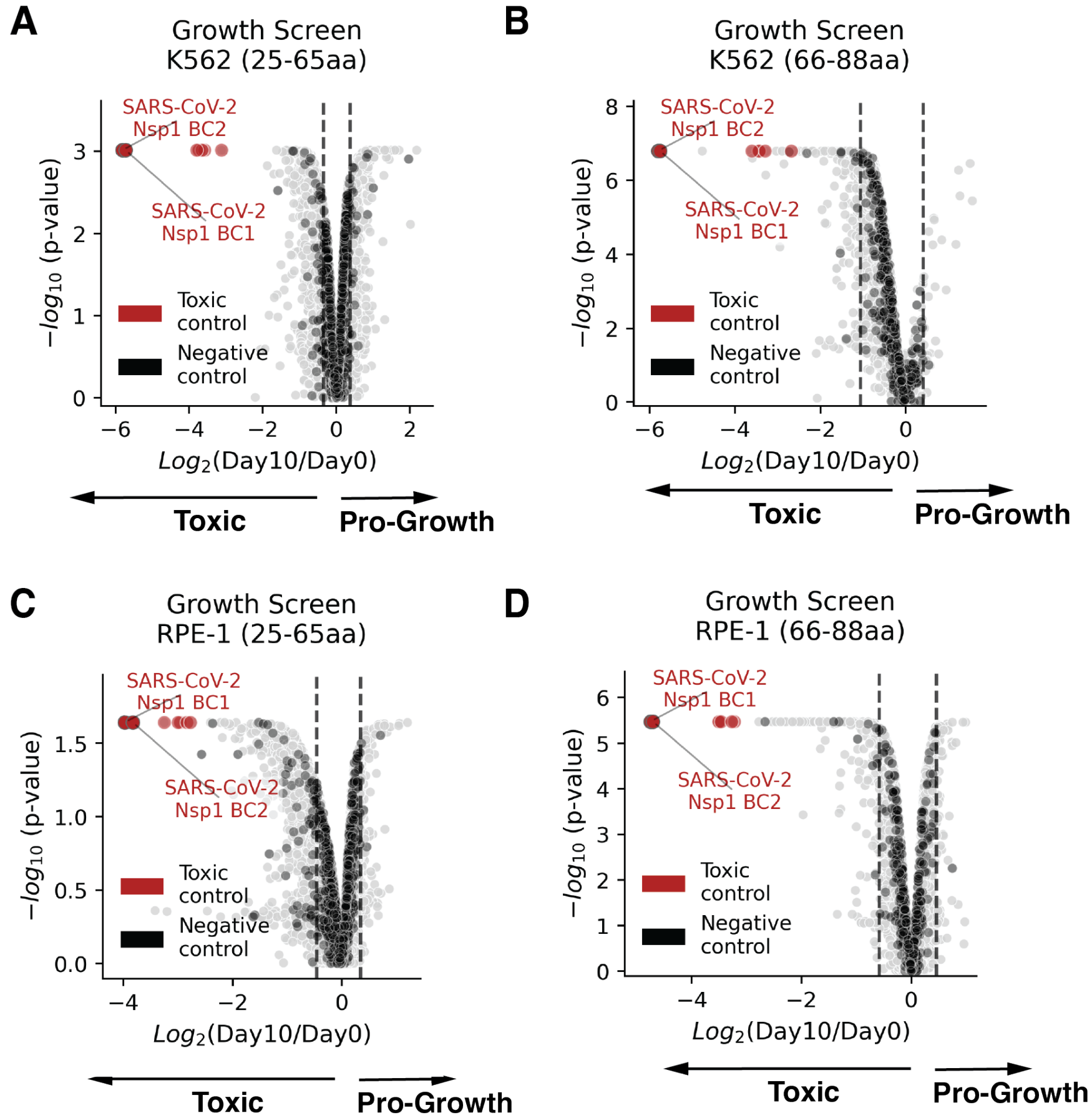

Fig. S5. Volcano plots of growth screens for viral smORFeome sublibraries 25-88aa in length. 10-day K562 screens with the (**A**) 25-65aa and (**B**) 66-88aa libraries, and RPE-1 screens with the (**C**) 25-65aa and (**D**) 66-88aa libraries.

bioRxiv preprint doi: https://doi.org/10.64898/2026.08.23.746553; this version posted August 24, 2026. The copyright holder for this preprint (which was not certified by peer review) is the author/funder, who has granted bioRxiv a license to display the preprint in perpetuity. It is made available under a CC-BY 4.0 International license.

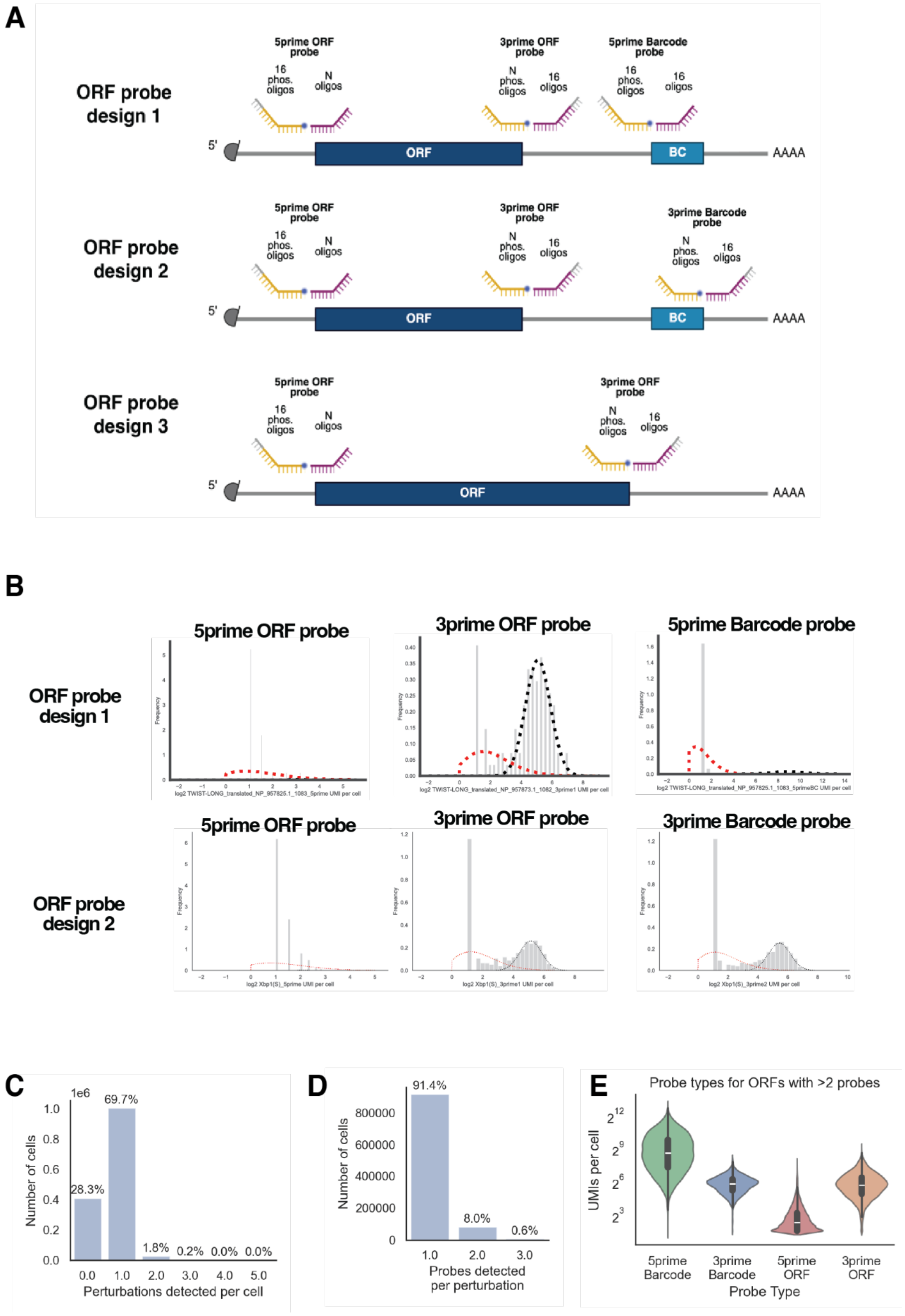

Fig. S6. Perturb-seq custom probe design and ORF calling. (**A**) Schematic of the barcoded ORF vector and probe design. (**B**) Example of mixed-model fitting of probe UMI counts for ORF assignment. Shown are two ORFs with three possible probe designs. (**C**) Number of smORF perturbations detected per cell. (**D**) Number of probes detected for cells with one perturbation. (**E**) Violin plots of UMI counts for the different probe types.

bioRxiv preprint doi: https://doi.org/10.64898/2026.08.23.746553; this version posted August 24, 2026. The copyright holder for this preprint (which was not certified by peer review) is the author/funder, who has granted bioRxiv a license to display the preprint in perpetuity. It is made available under a CC-BY 4.0 International license.

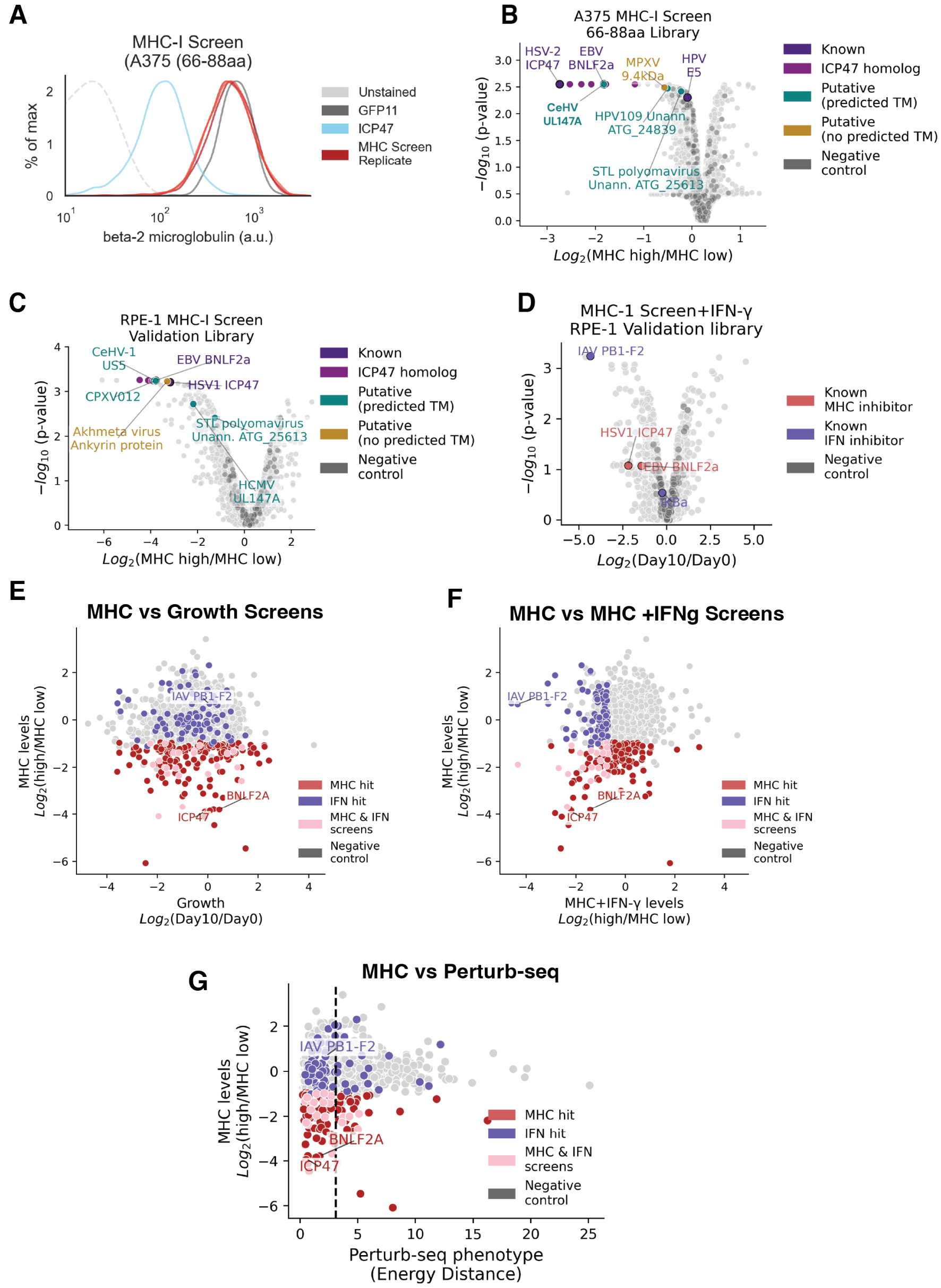

Fig. S7. MHC screen hits do not have growth or Perturb-seq phenotypes. (**A**) MHC-I levels measured by beta-2-microglobulin staining on A375 cells transduced with the 66-88aa viral smORF library, compared with positive (ICP47) and negative (GFP11) controls. Volcano plots of screen results for surface MHC-I levels in (**B**) A375 cells transduced with the 66-88aa viral smORF library and (**C**) RPE-1 cells transduced with the enriched library under basal conditions and (**D**) after 24 h of 200 ng/mL IFN-γ stimulation. Scatter plots of phenotypes from (**E**) the RPE-1 surface MHC-I screen versus the 10-day growth screen, (**F**) the RPE-1 surface MHC-I screen without versus with IFN-γ stimulation, and (**G**) the RPE-1 surface MHC-I screen versus the Perturb-seq phenotype score, calculated as the energy distance from negative controls.

bioRxiv preprint doi: https://doi.org/10.64898/2026.08.23.746553; this version posted August 24, 2026. The copyright holder for this preprint (which was not certified by peer review) is the author/funder, who has granted bioRxiv a license to display the preprint in perpetuity. It is made available under a CC-BY 4.0 International license.

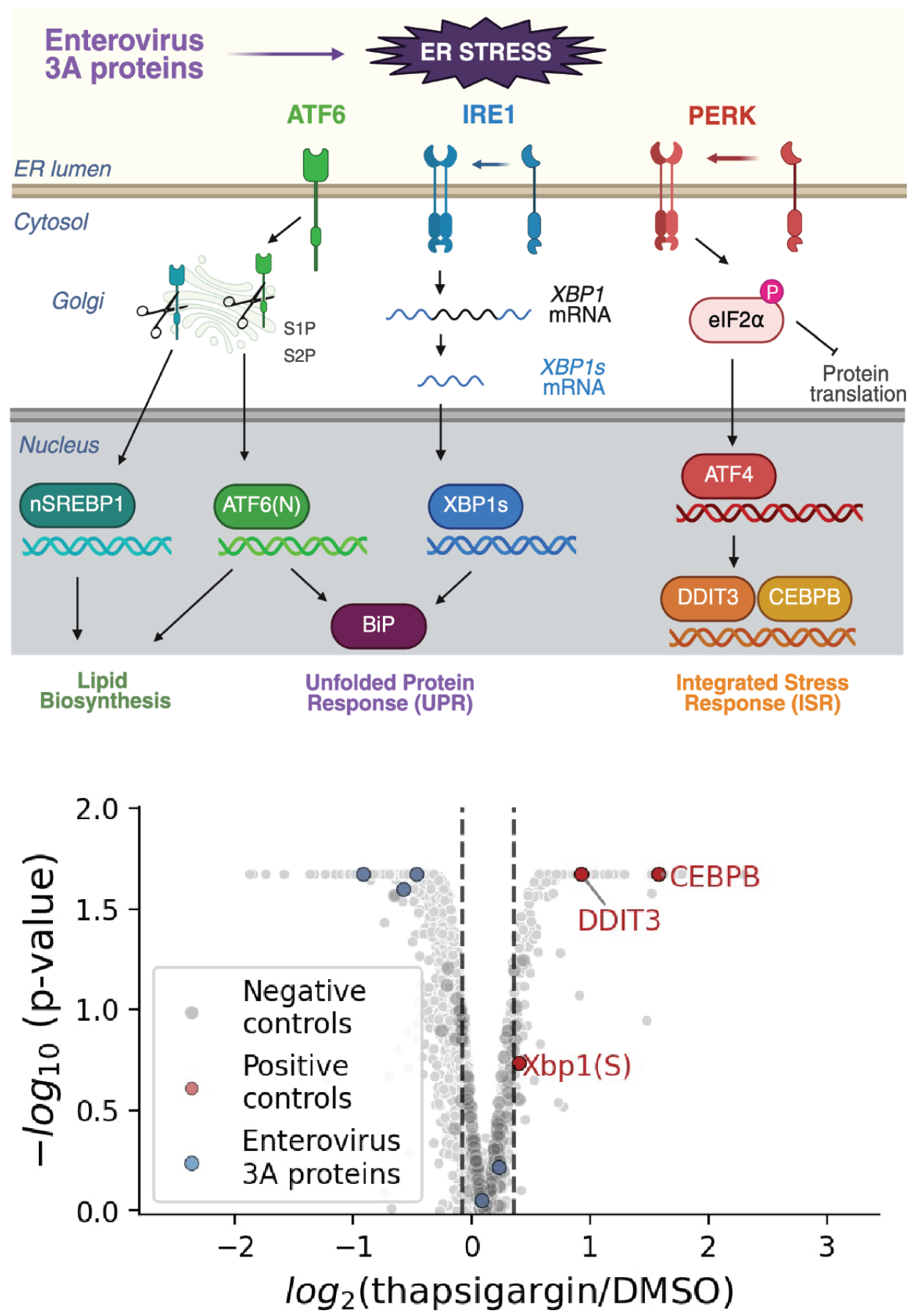

Fig. S8. Stress screens identify known and novel viral modulators of protein stress. (**A**) The unfolded protein response (UPR) pathway. (**B**) Volcano plot of the 4-day protein-stress growth screen under thapsigargin treatment compared with DMSO control. Positive controls relevant to the UPR are highlighted.

bioRxiv preprint doi: https://doi.org/10.64898/2026.08.23.746553; this version posted August 24, 2026. The copyright holder for this preprint (which was not certified by peer review) is the author/funder, who has granted bioRxiv a license to display the preprint in perpetuity. It is made available under a CC-BY 4.0 International license.

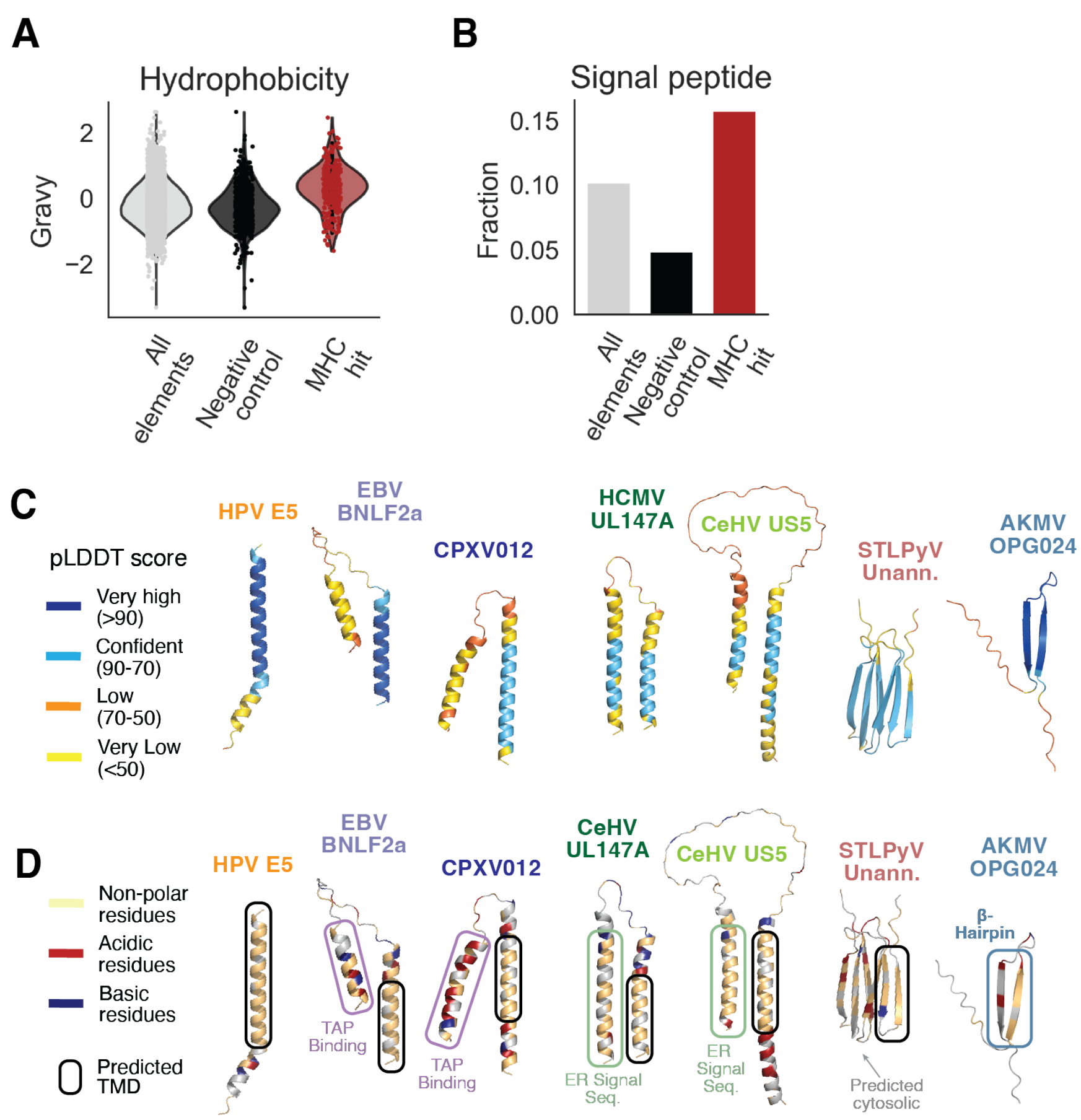

Fig. S9. Sequence and structures of MHC screen hits. (**A**) Violin plot of GRAVY hydrophobicity scores. (**B**) Fraction of elements with an ER signal-peptide sequence predicted by PHOBIUS. AF3-predicted structures of MHC-I inhibitors colored by (**C**) pLDDT score and (**D**) amino-acid property.

bioRxiv preprint doi: https://doi.org/10.64898/2026.08.23.746553; this version posted August 24, 2026. The copyright holder for this preprint (which was not certified by peer review) is the author/funder, who has granted bioRxiv a license to display the preprint in perpetuity. It is made available under a CC-BY 4.0 International license.

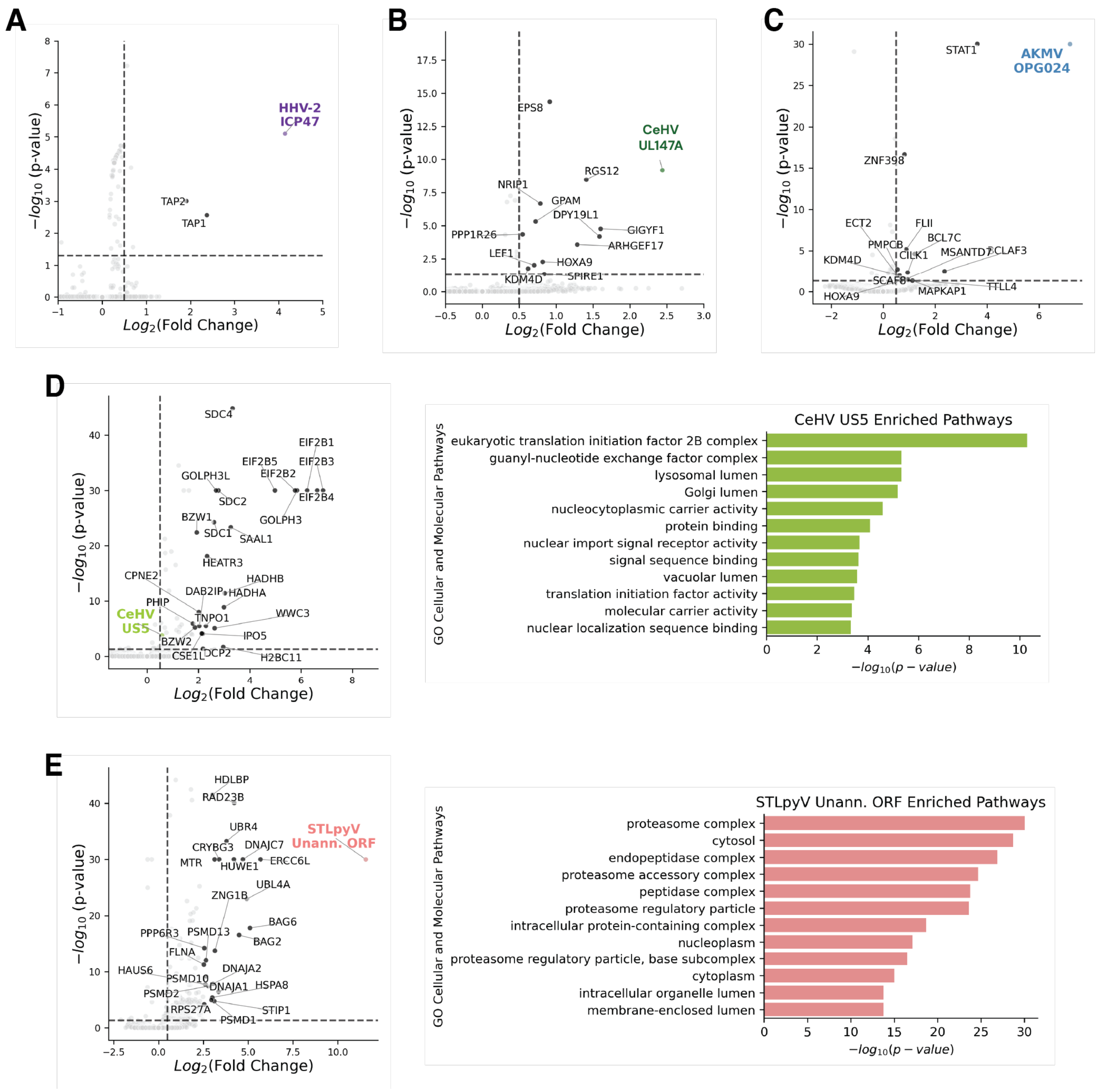

Fig. S10. Identification of host interactors of MHC inhibitors. Volcano plots of AE-MS performed on 293F cells transfected with C-term ALFA-tagged MHC inhibitors compared with ALFA-tagged mCherry or GFP controls for (**A**) HHV-2 ICP47, (**B**) CMV UL147A, (**C**) AKMV OPG024, (**D**) CeHV US5, and (**E**) an unannotated STLpyV smORF. GO pathway enrichment was also performed on all significant interactors of CeHV US5 and STLpyV smORF.

bioRxiv preprint doi: https://doi.org/10.64898/2026.08.23.746553; this version posted August 24, 2026. The copyright holder for this preprint (which was not certified by peer review) is the author/funder, who has granted bioRxiv a license to display the preprint in perpetuity. It is made available under a CC-BY 4.0 International license.

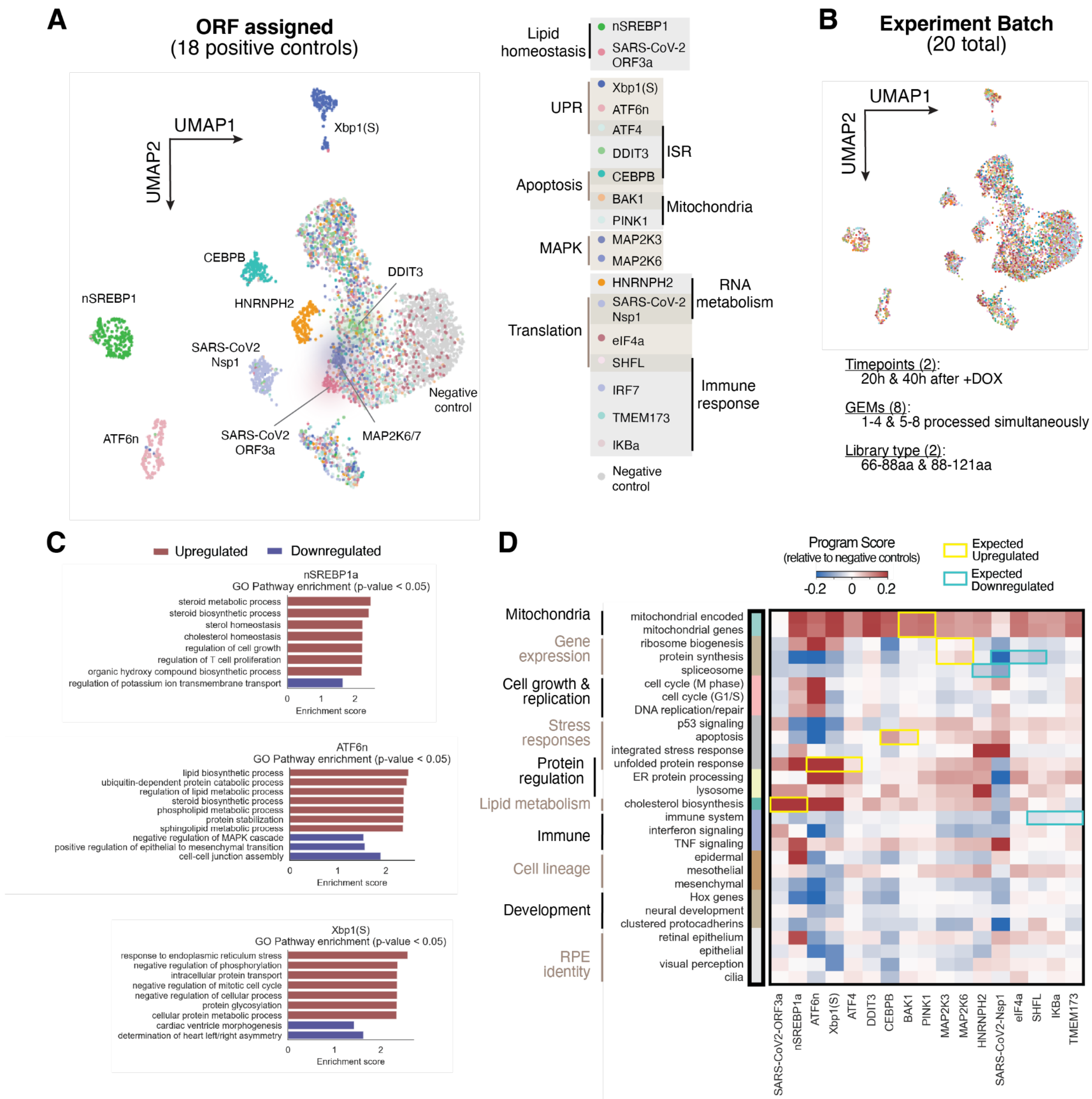

Fig. S11. Perturb-seq positive controls have expected phenotypes. UMAP of single cells assigned to positive and negative control ORFs, colored by (**A**) ORF assignment and (**B**) experiment batch, which includes batches divided up by timepoint, GEM group, and library type.
5 (**C**) Gene set enrichment analysis of all DEGs in example positive controls. (**D**) Heatmap of program scores derived from RPE-1 essential-gene Perturb-seq relative to negative controls.

bioRxiv preprint doi: https://doi.org/10.64898/2026.08.23.746553; this version posted August 24, 2026. The copyright holder for this preprint (which was not certified by peer review) is the author/funder, who has granted bioRxiv a license to display the preprint in perpetuity. It is made available under a CC-BY 4.0 International license.

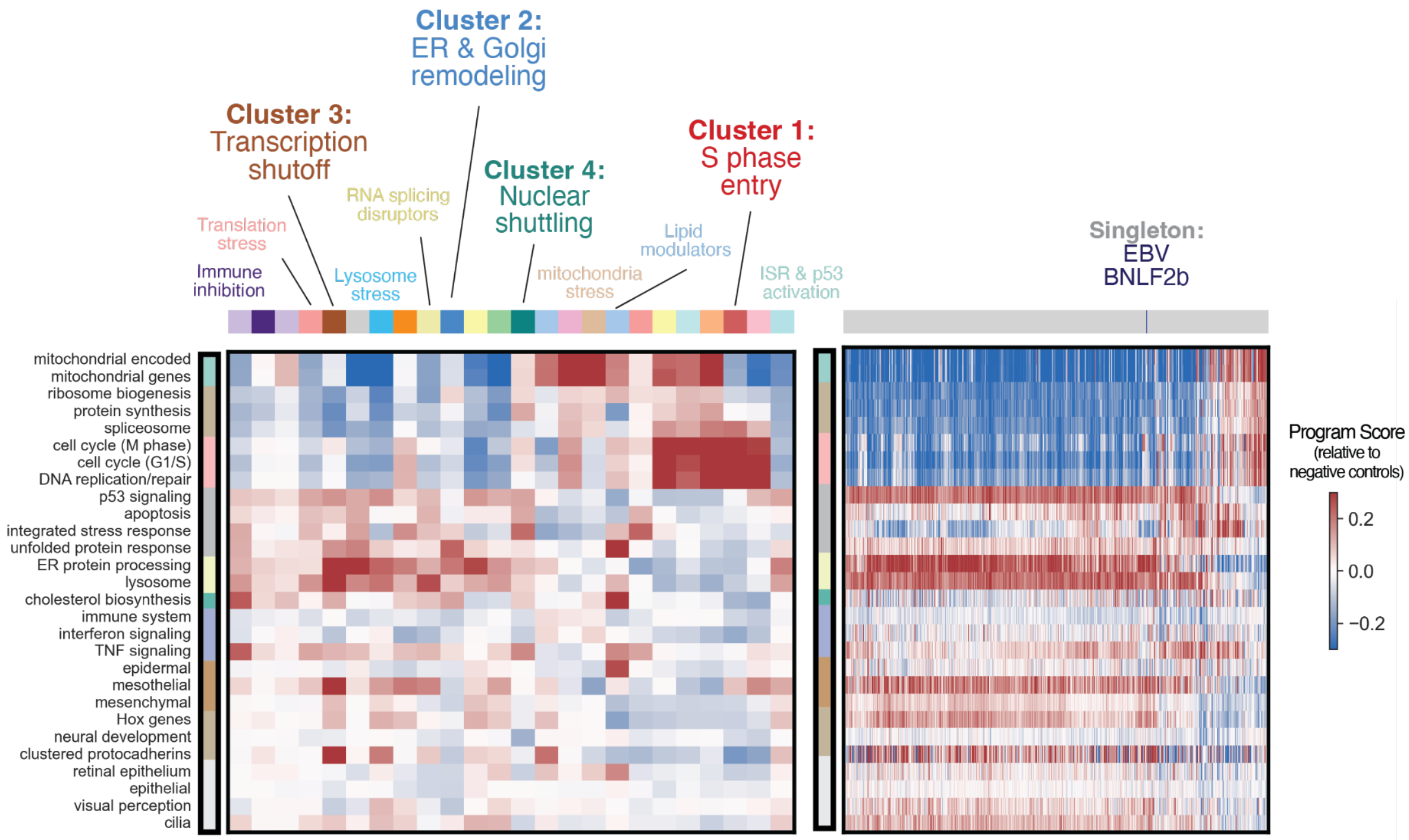

Fig. S12. Gene pathway scoring from transcriptional profiles. Heatmap of program scores for (Left) Perturb-seq clusters and (Right) singletons that pass the energy-distance test, with EBV BNLF2b marked.

bioRxiv preprint doi: https://doi.org/10.64898/2026.08.23.746553; this version posted August 24, 2026. The copyright holder for this preprint (which was not certified by peer review) is the author/funder, who has granted bioRxiv a license to display the preprint in perpetuity. It is made available under a CC-BY 4.0 International license.

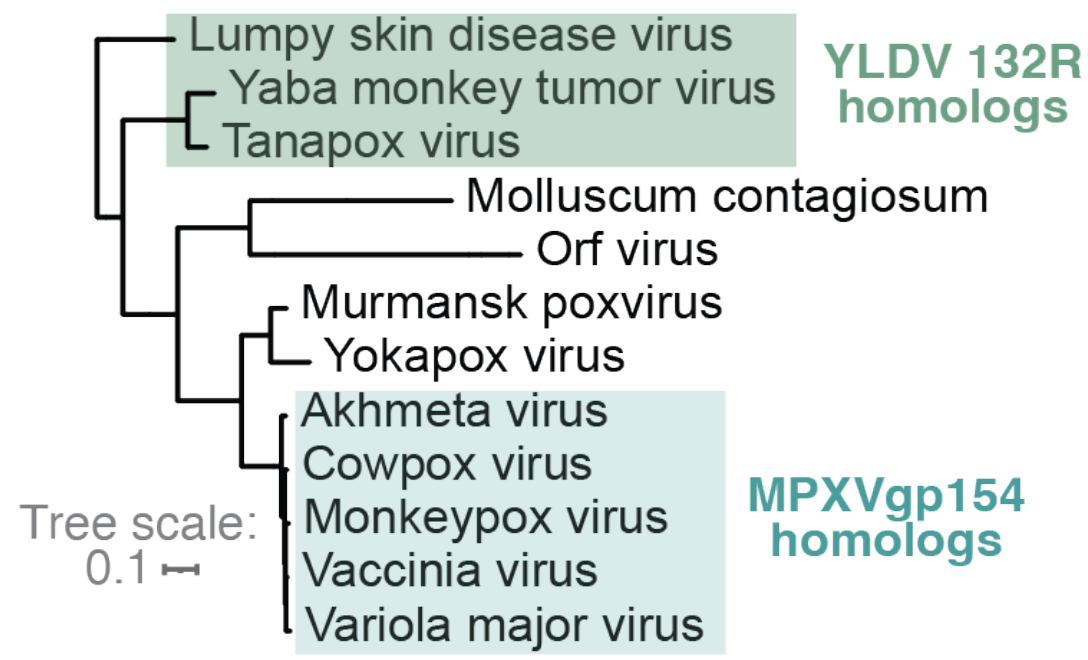

Fig. S13. Phylogenetic tree of poxvirus lineage. Trees were obtained from ICTV with presence of MPXVgp154 and YLDV 132R homologs highlighted.

bioRxiv preprint doi: https://doi.org/10.64898/2026.08.23.746553; this version posted August 24, 2026. The copyright holder for this preprint (which was not certified by peer review) is the author/funder, who has granted bioRxiv a license to display the preprint in perpetuity. It is made available under a CC-BY 4.0 International license.

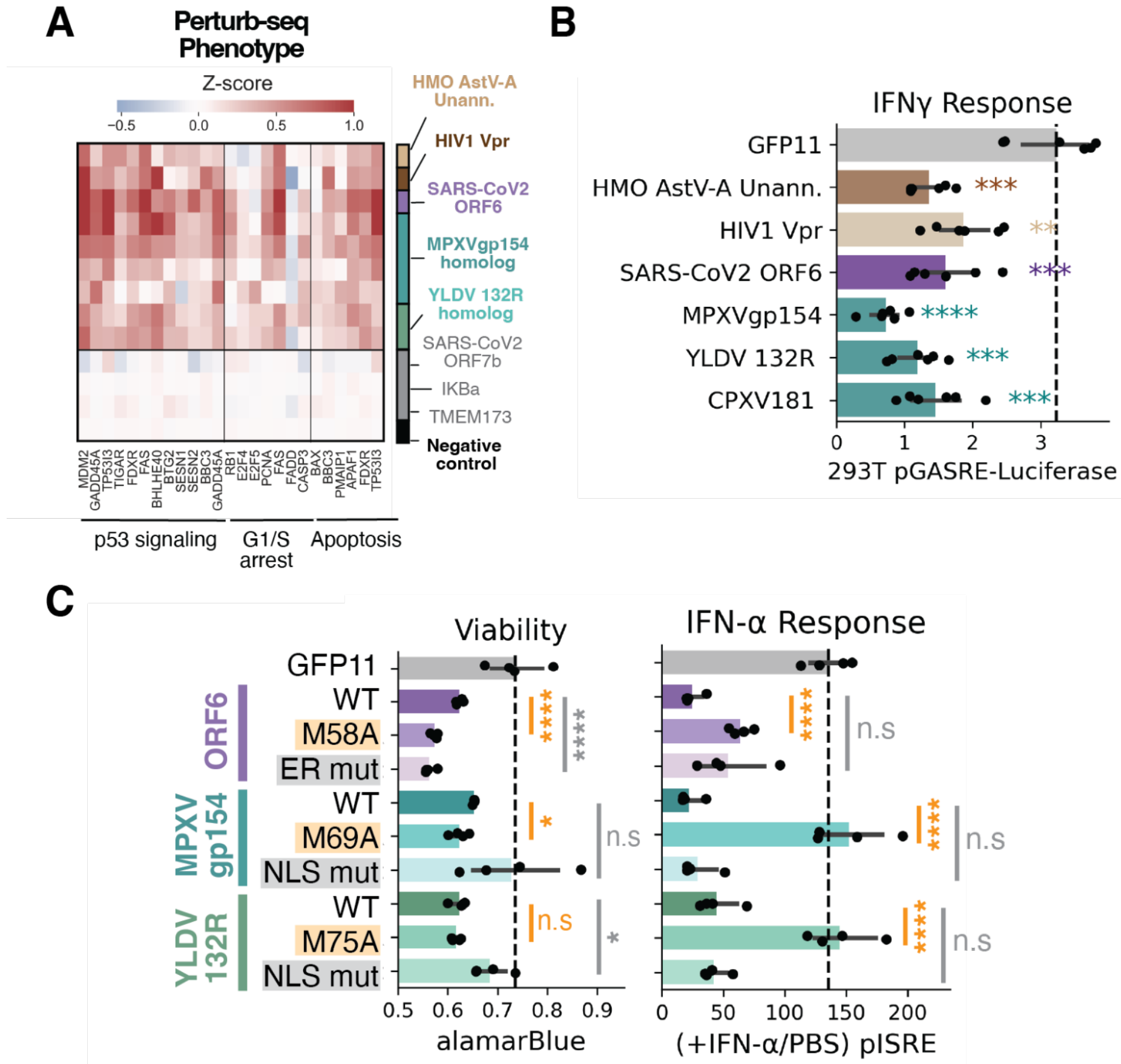

Fig. S14. Phenotypic characterization of cluster 4, the nuclear shuttling inhibitors. (**A**) Heatmap of DEGs grouped by pathway. (**B**) IFN-γ response upon overexpression in HEK293T cells, measured with a pGAS-RE-NanoLuc assay after 16 h of stimulation with 200 ng/mL IFN-γ. (**C**) HEK293T cells transfected with the indicated WT and mutant ORFs were examined for viability (left) by alamarBlue assay and for IFN-α response (right) by pISRE-NanoLuc assay after 16 h of stimulation with 75 ng/mL IFN-α.

bioRxiv preprint doi: https://doi.org/10.64898/2026.08.23.746553; this version posted August 24, 2026. The copyright holder for this preprint (which was not certified by peer review) is the author/funder, who has granted bioRxiv a license to display the preprint in perpetuity. It is made available under a CC-BY 4.0 International license.

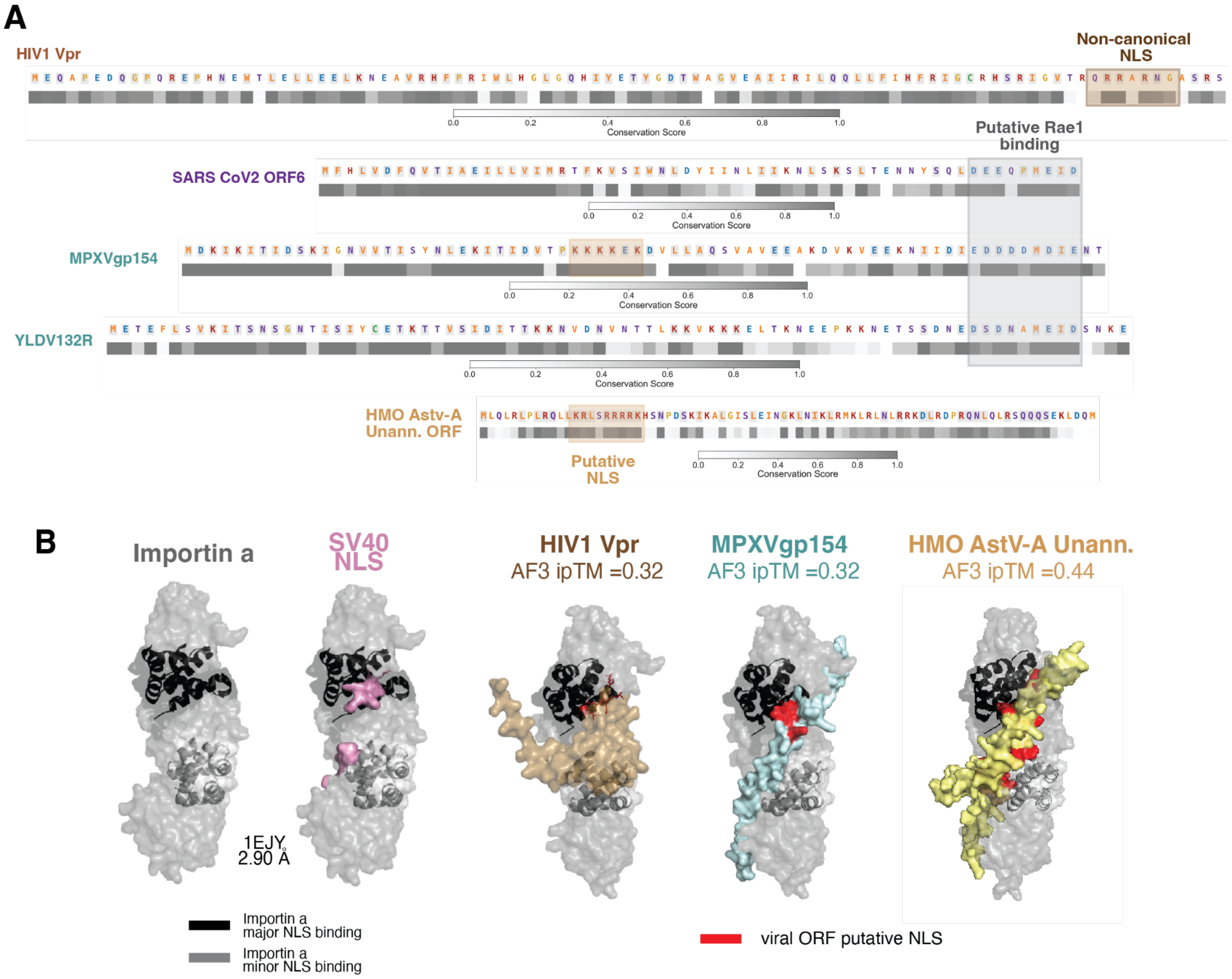

Fig. S15. Identification of putative NLSs in nuclear shuttling inhibitor cluster elements. (**A**) Sequence analysis and conservation scores of cluster elements, with putative NLS and putative Rae1-binding motifs highlighted. (**B**) Comparison of importin-α bound to the SV40 NLS (PDB: 1EJY) with AF3-predicted structures of importin-α bound to indicated ORFs.

bioRxiv preprint doi: https://doi.org/10.64898/2026.08.23.746553; this version posted August 24, 2026. The copyright holder for this preprint (which was not certified by peer review) is the author/funder, who has granted bioRxiv a license to display the preprint in perpetuity. It is made available under a CC-BY 4.0 International license.

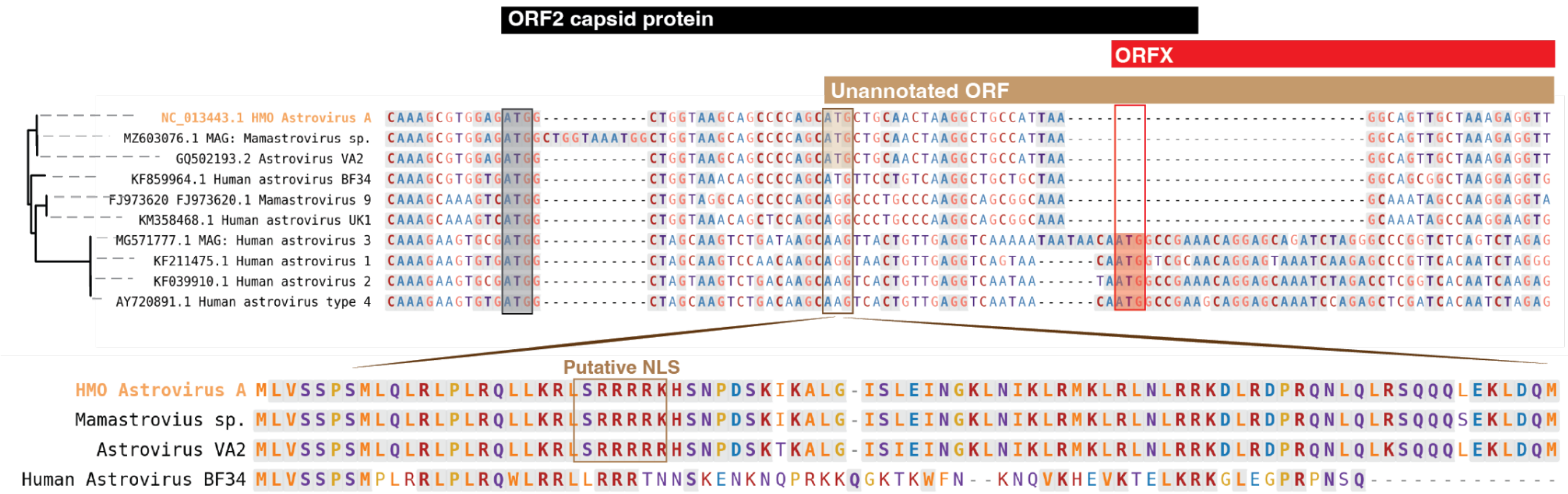

Fig. S16. Astrovirus genome locus and protein sequences of the HMO AstV-A unannotated ORF across lineages. MSA of astrovirus genomic loci containing HMO AstV-A Unann. ORF with its +1 start codon highlighted, and comparison of the amino-acid sequences of smORFs sharing this +1 start codon.

bioRxiv preprint doi: https://doi.org/10.64898/2026.08.23.746553; this version posted August 24, 2026. The copyright holder for this preprint (which was not certified by peer review) is the author/funder, who has granted bioRxiv a license to display the preprint in perpetuity. It is made available under a CC-BY 4.0 International license.

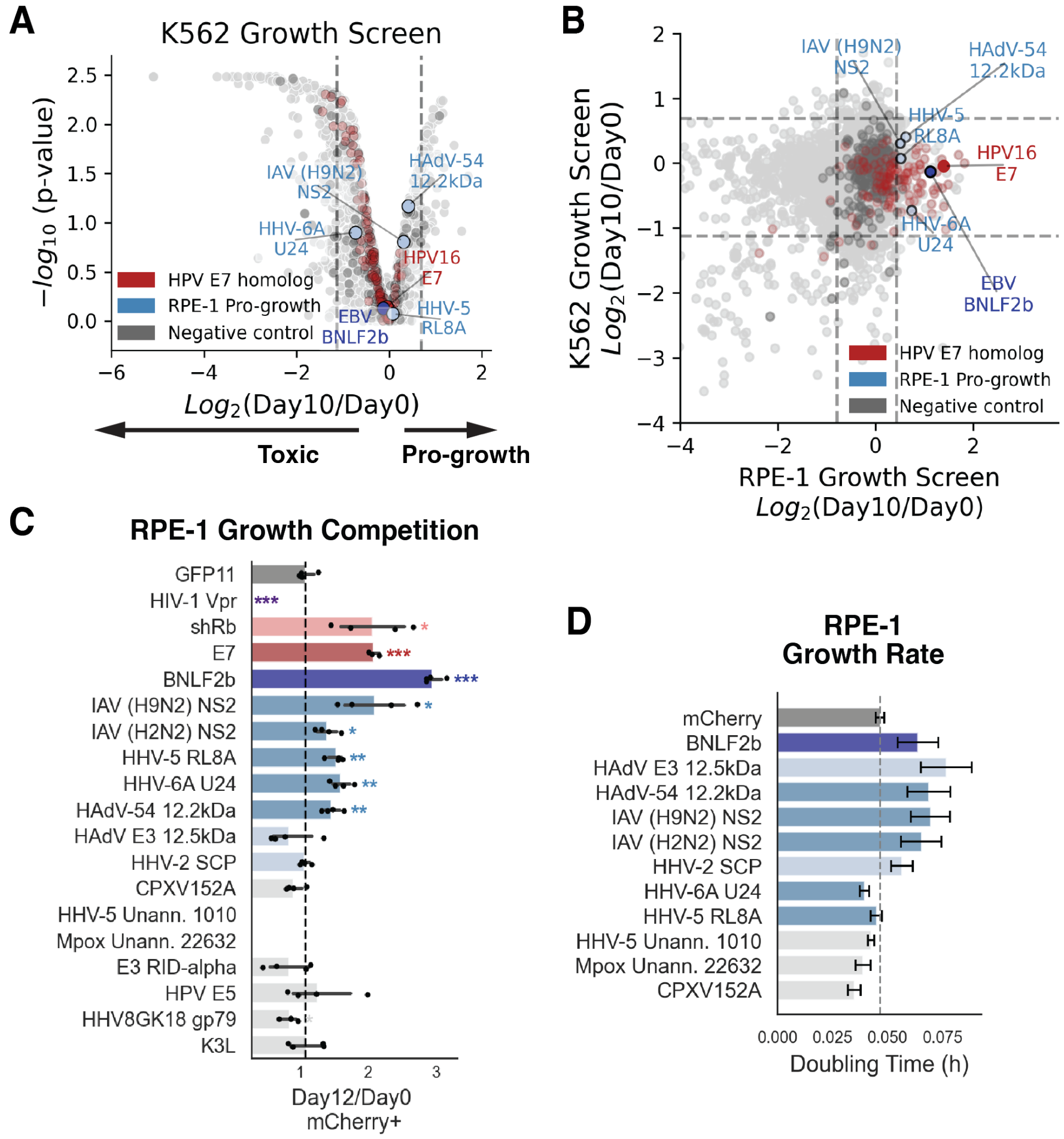

Fig. S17. RPE-1 pro-growth hits do not promote growth in K562 cells but validate in RPE-1 cells. (**A**) Volcano plot of the 10-day K562 growth screen with pro-growth hits highlighted. (**B**) Scatter plot of K562 versus RPE-1 growth screen phenotypes with pro-growth hits highlighted. (**C**) Arrayed growth-competition and (**D**) arrayed growth-rate measurements by confluency tracking of RPE-1 cells overexpressing the indicated ORFs.

bioRxiv preprint doi: https://doi.org/10.64898/2026.08.23.746553; this version posted August 24, 2026. The copyright holder for this preprint (which was not certified by peer review) is the author/funder, who has granted bioRxiv a license to display the preprint in perpetuity. It is made available under a CC-BY 4.0 International license.

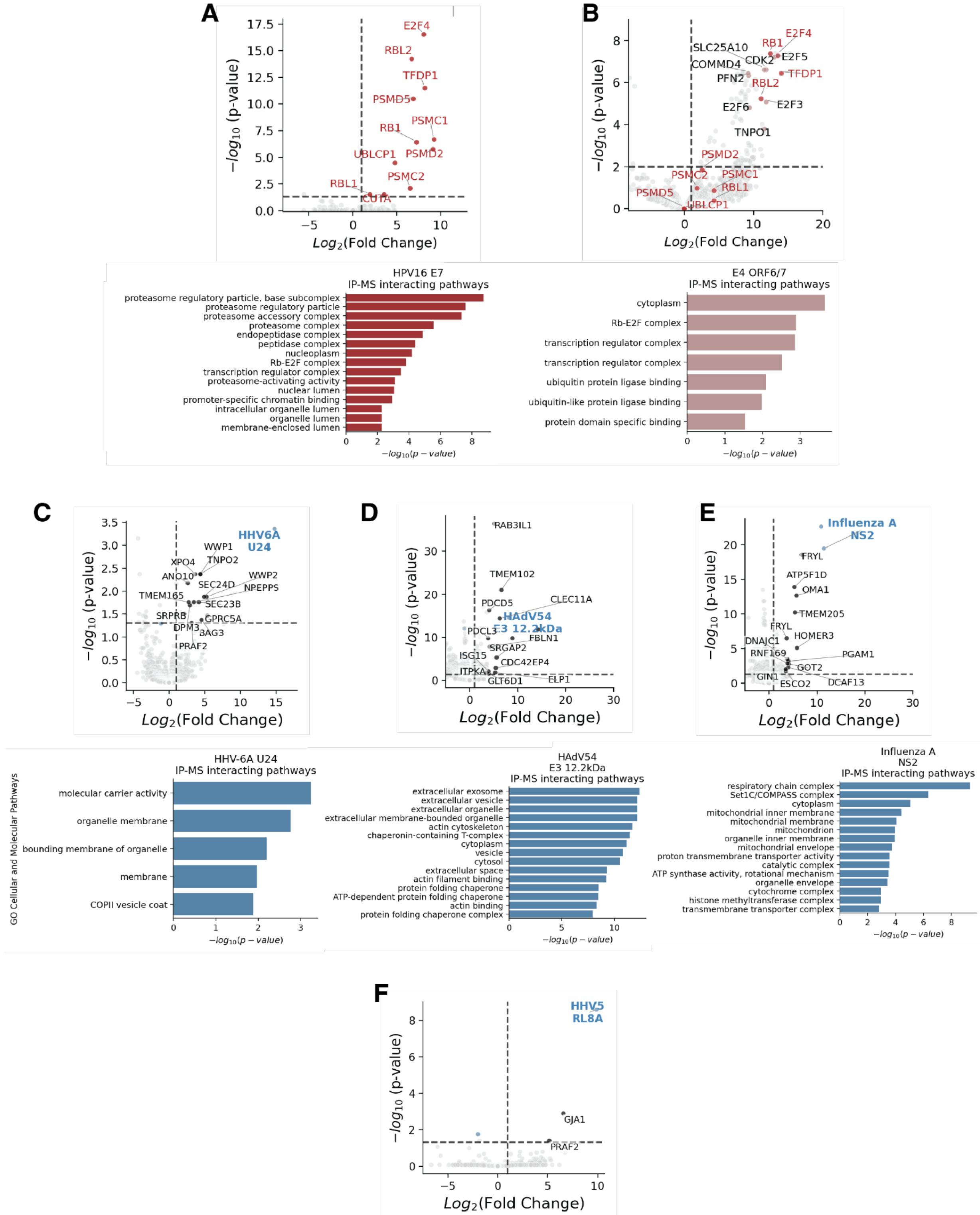

Fig. S18. Identification of host interactors of validated RPE-1 pro-growth hits. Volcano plots of AE-MS performed on RPE-1 cells overexpressing ALFA-tagged pro-growth hits compared with ALFA-tagged mCherry or GFP controls, and GO pathway enrichment of significant interactors, for (**A**) HPV16 E7, (**B**) HAdV E4 ORF6/7, (**C**) HHV-6A U24, (**D**) HAdV54 E3 12.2 kDa, (**E**) IAV NS2, and (F) HHV-5 RL8A.

bioRxiv preprint doi: https://doi.org/10.64898/2026.08.23.746553; this version posted August 24, 2026. The copyright holder for this preprint (which was not certified by peer review) is the author/funder, who has granted bioRxiv a license to display the preprint in perpetuity. It is made available under a CC-BY 4.0 International license.

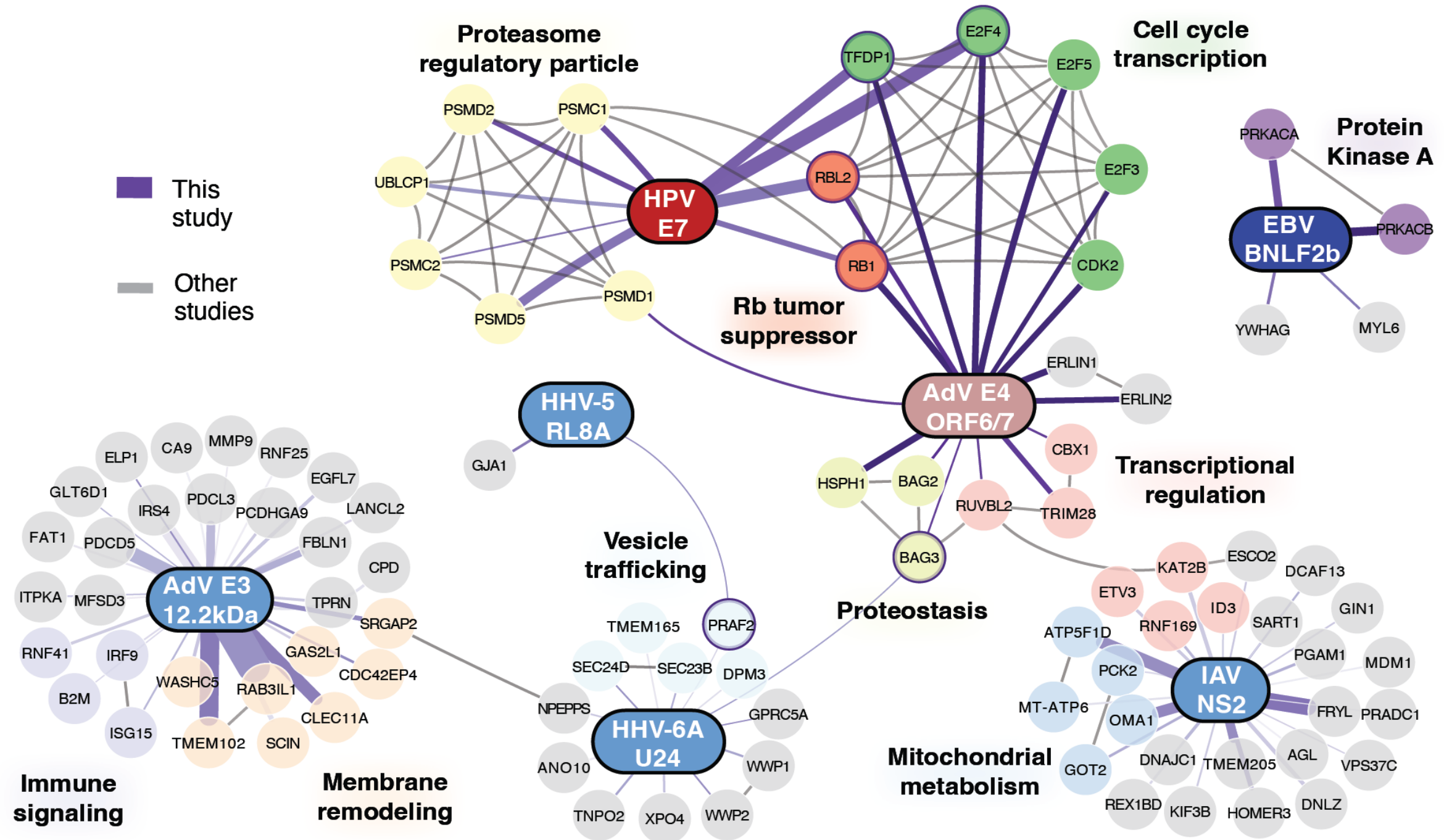

Fig. S19. Protein interaction networks of pro-growth hits align with Perturb-seq cluster and singleton assignments. Networks were generated by integrating our AE-MS interactors with host protein networks derived from STRING, with edges drawn if the interaction confidence score is >0.75. Functions of host-interactor groups were manually annotated.

bioRxiv preprint doi: https://doi.org/10.64898/2026.08.23.746553; this version posted August 24, 2026. The copyright holder for this preprint (which was not certified by peer review) is the author/funder, who has granted bioRxiv a license to display the preprint in perpetuity. It is made available under a CC-BY 4.0 International license.

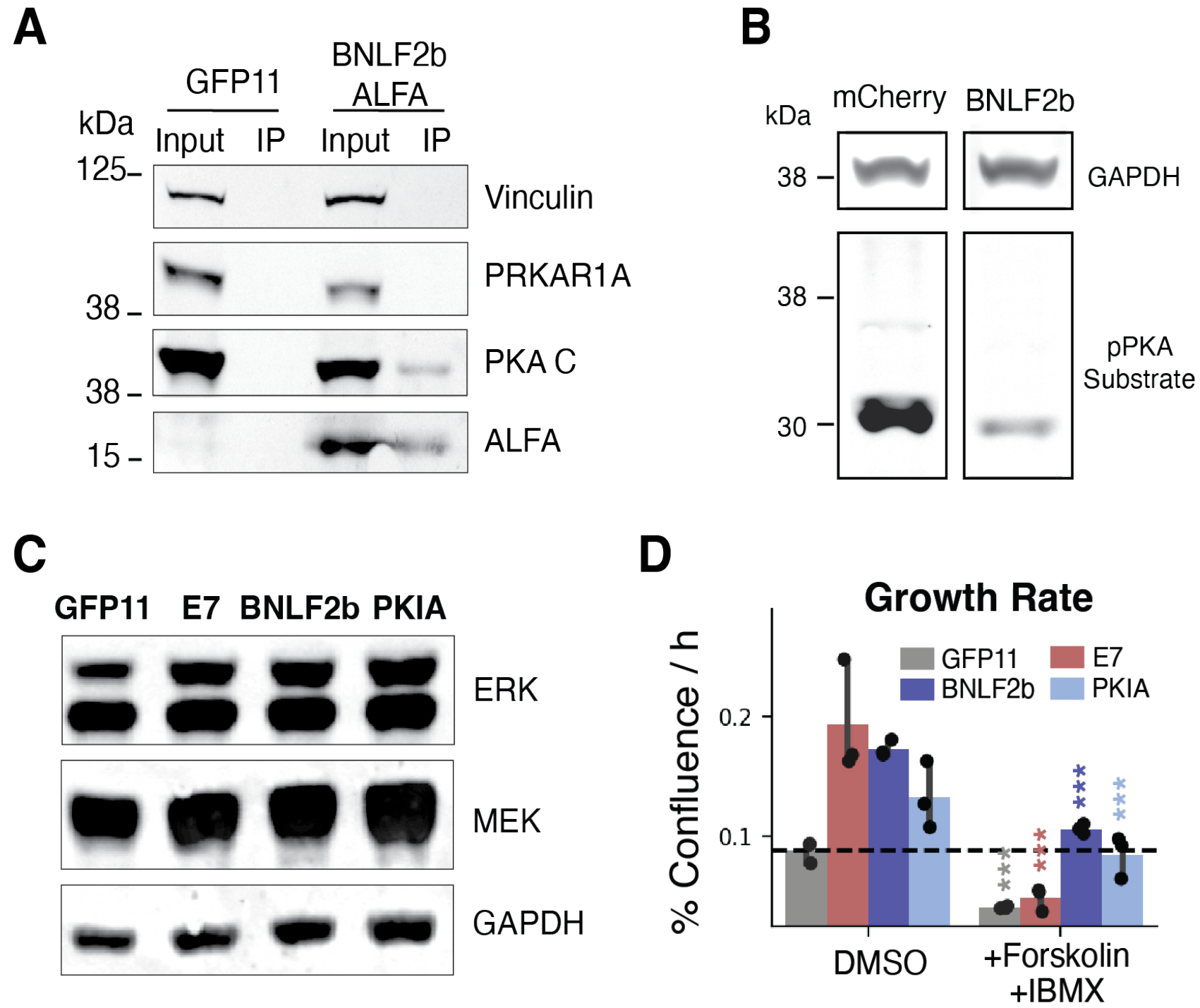

Fig. S20. Further characterization of BNLF2b modulation of PKA and MAPK. (**A**) IP-Western of A375 cells overexpressing ALFA-tagged BNLF2b for examining interaction with PRKAR1A and PKA Cα (PRKACA). (**B**) Western blot of pPKA substrate levels in mCherry and BNLF2b overexpressing RPE-1 cells. (**C**) Total ERK and MEK levels of indicated ORFs overexpressed in RPE-1. (**D**) Growth rates of RPE-1 cells overexpressing the indicated ORFs, grown either in DMSO vehicle-control medium or in medium containing forskolin and IBMX.

bioRxiv preprint doi: https://doi.org/10.64898/2026.08.23.746553; this version posted August 24, 2026. The copyright holder for this preprint (which was not certified by peer review) is the author/funder, who has granted bioRxiv a license to display the preprint in perpetuity. It is made available under a CC-BY 4.0 International license.

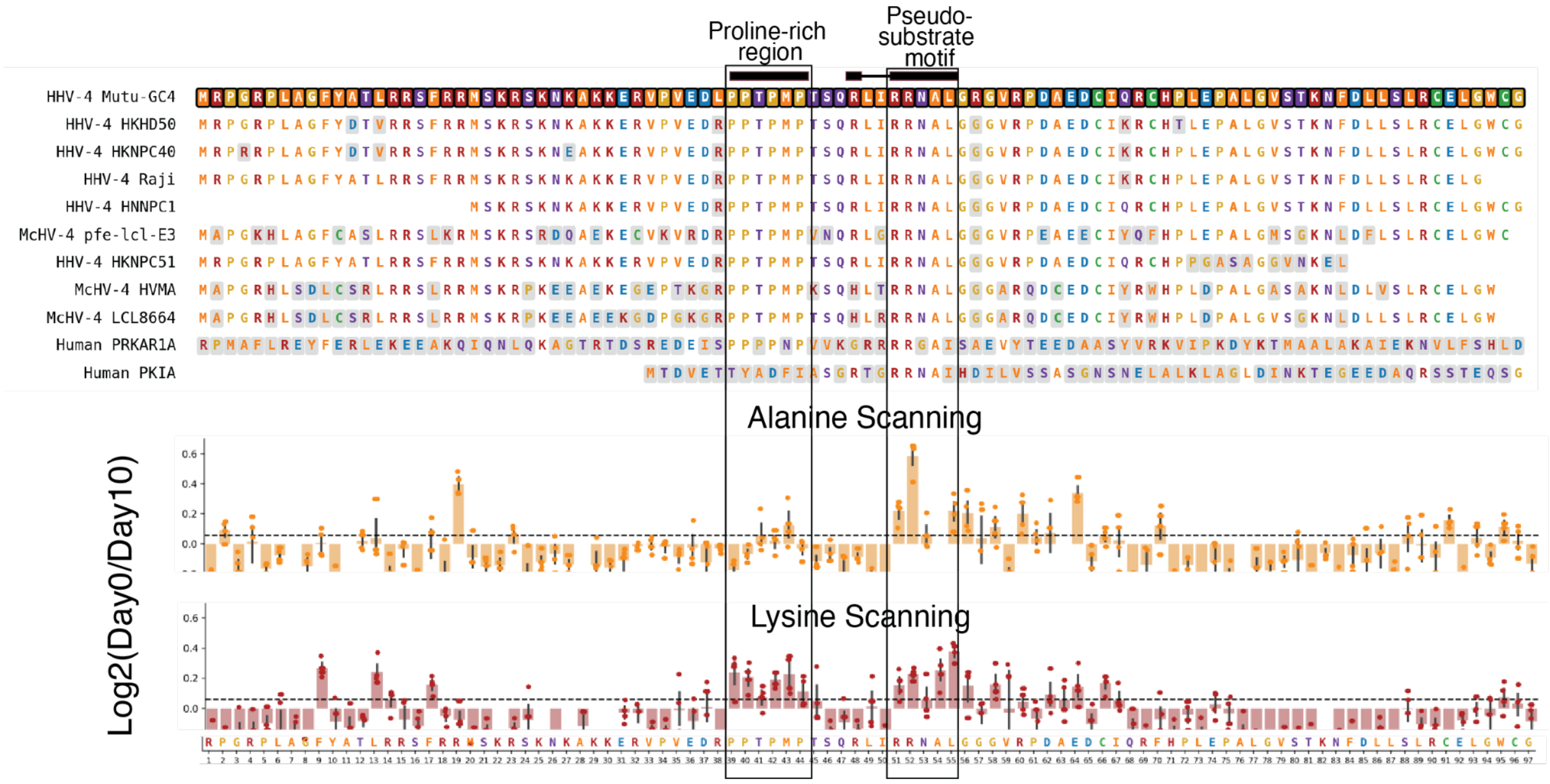

Fig. S21. BNLF2b mutagenesis screens identify key BNLF2b residues that impact growth. (Top) MSA of BNLF2b homologs. Residues are aligned with the results of (Middle) alanine and (Bottom) lysine scanning from 10-day RPE-1 growth screens.

bioRxiv preprint doi: https://doi.org/10.64898/2026.08.23.746553; this version posted August 24, 2026. The copyright holder for this preprint (which was not certified by peer review) is the author/funder, who has granted bioRxiv a license to display the preprint in perpetuity. It is made available under a CC-BY 4.0 International license.

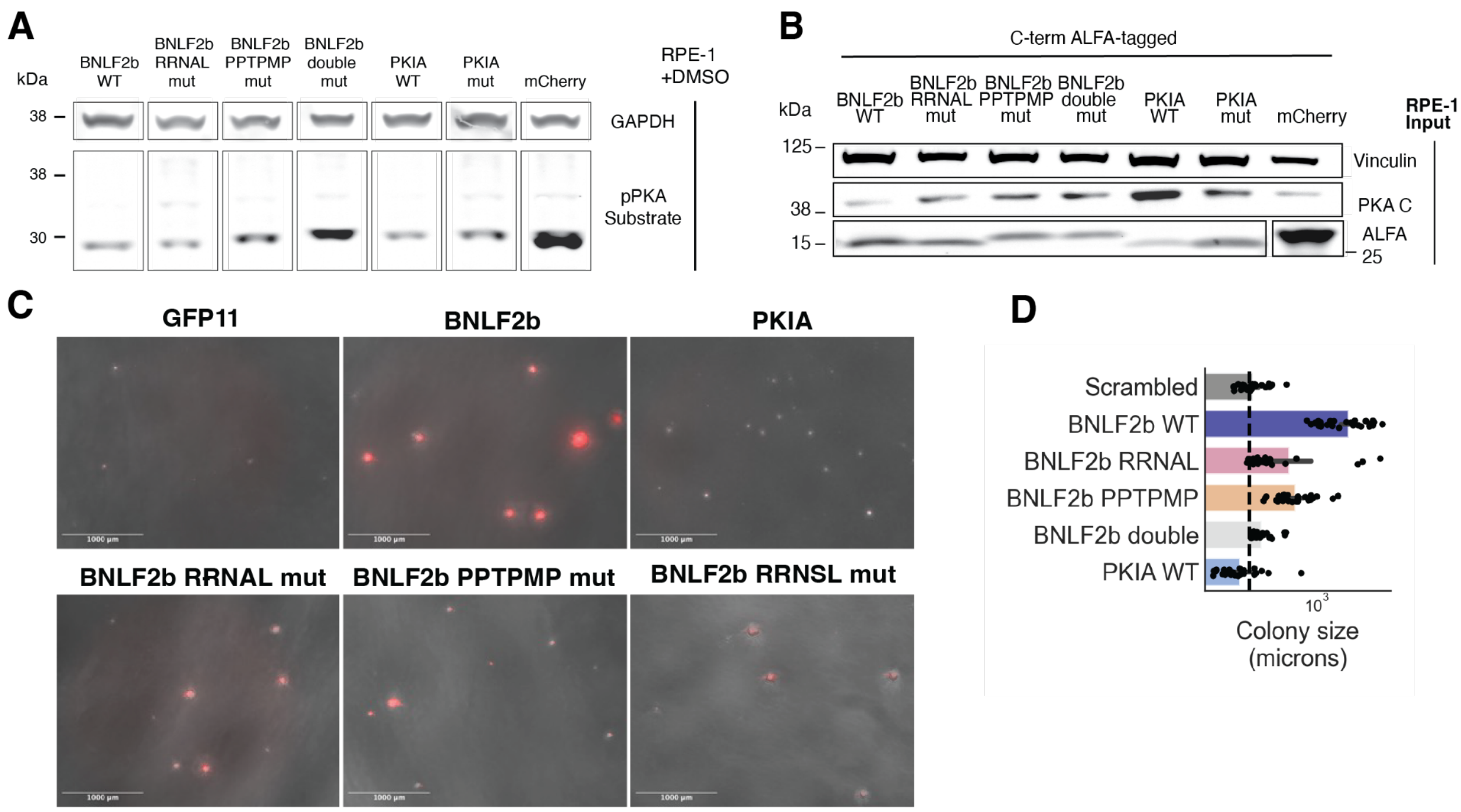

Fig. S22. Further characterization of BNLF2b mutants. (**A**) pPKA substrate levels in RPE-1 cells overexpressing the indicated ORFs. (**B**) Input for the IP-MS of ALFA-tagged BNLF2b and PKIA (WT and mutants). (**C**) Soft-agar transformation assay 45 days after seeding of RPE-1 cells lentivirally transduced with constitutive overexpression constructs for the indicated ORFs. Images show phase contrast overlaid with mCherry fluorescence. (**D**) Quantification of colony sizes.

bioRxiv preprint doi: https://doi.org/10.64898/2026.08.23.746553; this version posted August 24, 2026. The copyright holder for this preprint (which was not certified by peer review) is the author/funder, who has granted bioRxiv a license to display the preprint in perpetuity. It is made available under a CC-BY 4.0 International license.

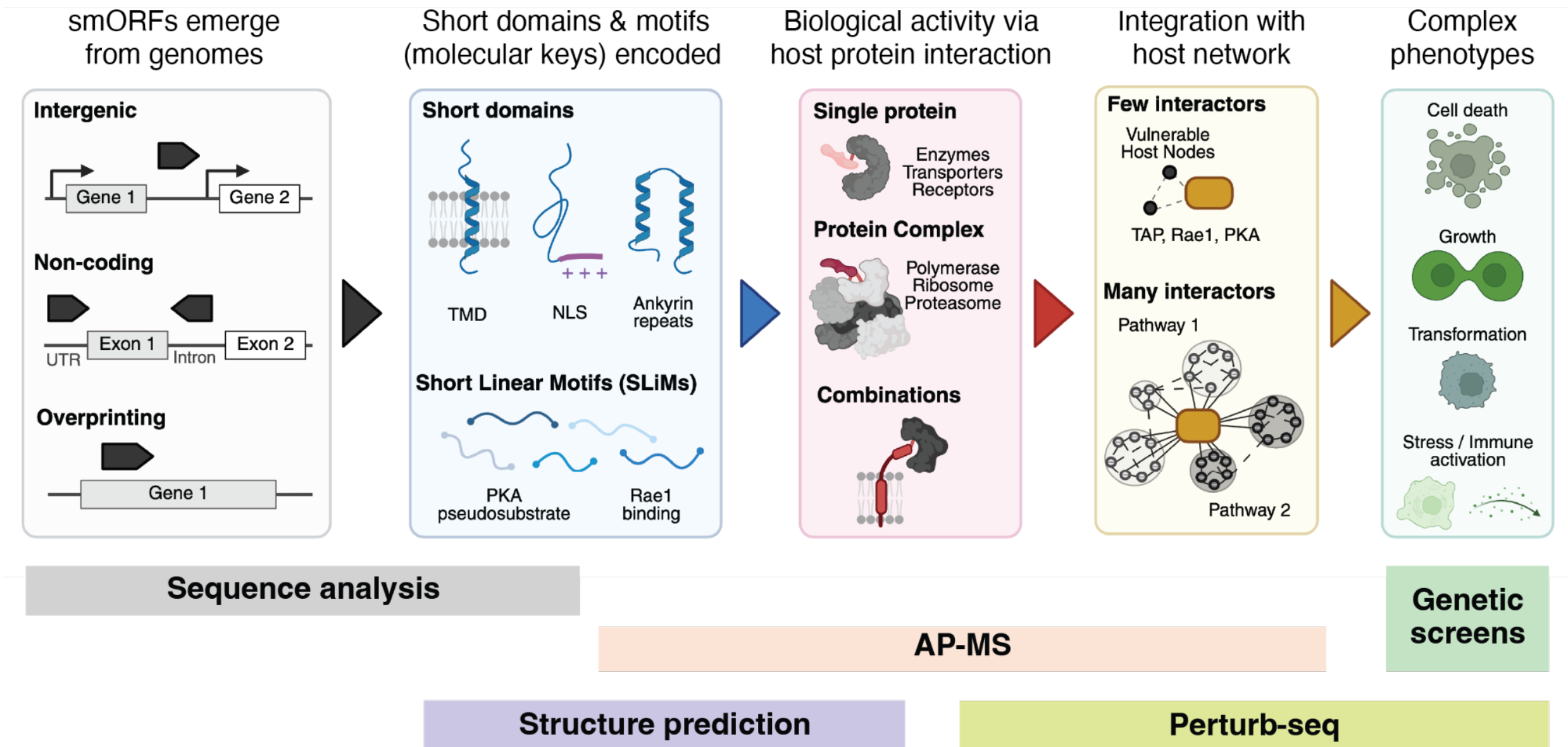

Fig. S23. A model for emergence of functional smORFs. smORFs emerge in viral genomes in intergenic or non-coding regions as well as overlapping in alternative frames from other coding sequences by overprinting. smORFs generate microproteins containing short domains or SLiMs. Molecular keys enable interactions with host proteins and give rise to biological activities. Combinations of keys further enhance potency or give rise to multifunctionality. Integration with the host protein network modulates host pathways that result in emergence of complex cellular phenotypes.

bioRxiv preprint doi: https://doi.org/10.64898/2026.08.23.746553; this version posted August 24, 2026. The copyright holder for this preprint (which was not certified by peer review) is the author/funder, who has granted bioRxiv a license to display the preprint in perpetuity. It is made available under a CC-BY 4.0 International license.

Table S1. Antibodies used for western blotting and flow cytometry.

Target	Supplier	Supplier Catalog #	WB dilution	Flow cytometry dilution
ALFA-tag	Nano-Tag	N1582	1:5000	
β2M	Biolegend	316312		1:100
Mek1/2	Cell Signaling Technology	9122S	1:1000	
Phospho-MEK1/2 (Ser217/221)	Cell Signaling Technology	9121S	1:1000	
Erk1/2	Cell Signaling Technology	4695S	1:1000	
Phospho-p44/42 MAPK (Erk1/2) (Thr202/Tyr204)	Cell Signaling Technology	4370S	1:1000	
Phospho-PKA Substrate (RRXS*/T*) (100G7E)	Cell Signaling Technology	9624S	1:1000	
GAPDH (14C10)	Cell Signaling Technology	2118L	1:5000	
Vinculin	Cell Signaling Technology	13901	1:2000	
PKA C-α	Cell Signaling Technology	4782	1:1000	
Rb	Cell Signaling Technology	9309	1:1000	
E2F1	Cell Signaling Technology	3742	1:1000	
E2F4	Cell Signaling Technology	40291	1:1000	
DP-1	Santa Cruz Biotechnology	sc-53642	1:500	
pStat1	Cell Signaling Technology	9167		
E-Cadherin	Cell Signaling Technology	3195	1:1000	
E-Cadherin	R&D Systems	AF748	N/A	
Fibronectin	Santa Cruz Biotechnology	sc-8422	1:500	
Vimentin	Santa Cruz Biotechnology	sc-6260	1:500	
mCD19	Thermo Scientific	11-0191-82	1:100	

bioRxiv preprint doi: https://doi.org/10.64898/2026.08.23.746553; this version posted August 24, 2026. The copyright holder for this preprint (which was not certified by peer review) is the author/funder, who has granted bioRxiv a license to display the preprint in perpetuity. It is made available under a CC-BY 4.0 International license.

Table S2. Primers for genetic screens.

Target	Primer Name	Sequence
Primers for NGS library preparation for 50K viral smORF and Enriched library screens (PCR1)	oYC858_pINDUCER_fw_1N	ACACTCTTTCCCTACACGACGCTCTTCCGATCTNCCAGTAGGTCCACTATGAGT
	oYC859_pINDUCER_fw_2N	ACACTCTTTCCCTACACGACGCTCTTCCGATCTNNCCAGTAGGTCCACTATGAGT
	oYC860_pINDUCER_fw_3N	ACACTCTTTCCCTACACGACGCTCTTCCGATCTNNNCCAGTAGGTCCACTATGAGT
	oYC861_pINDUCER_fw_4N	ACACTCTTTCCCTACACGACGCTCTTCCGATCTNNNNCCAGTAGGTCCACTATGAGT
	oYC862_pINDUCER_fw_5N	ACACTCTTTCCCTACACGACGCTCTTCCGATCTNNNNNCCAGTAGGTCCACTATGAGT
	oYC863_pINDUCER_fw_6N	ACACTCTTTCCCTACACGACGCTCTTCCGATCTNNNNNNCCAGTAGGTCCACTATGAGT
	oYC864_pINDUCER_rv_1N	TGGAGTTCAGACGTGTGCTCTTCCGATCTNCTAGTTCCGCTTACACAGCT
	oYC865_pINDUCER_rv_2N	TGGAGTTCAGACGTGTGCTCTTCCGATCTNNCTAGTTCCGCTTACACAGCT
	oYC866_pINDUCER_rv_3N	TGGAGTTCAGACGTGTGCTCTTCCGATCTNNNCTAGTTCCGCTTACACAGCT
	oYC867_pINDUCER_rv_4N	TGGAGTTCAGACGTGTGCTCTTCCGATCTNNNNCTAGTTCCGCTTACACAGCT
	oYC868_pINDUCER_rv_5N	TGGAGTTCAGACGTGTGCTCTTCCGATCTNNNNNCTAGTTCCGCTTACACAGCT
	oYC869_pINDUCER_rv_6N	TGGAGTTCAGACGTGTGCTCTTCCGATCTNNNNNNCTAGTTCCGCTTACACAGCT
Primers for NGS library preparation for BNLF2b alanine and lysine mutational scanning screens (PCR1)	oYC910_BNLF2b-DMS_fw-1N_tm61	ACACTCTTTCCCTACACGACGCTCTTCCGATCTNatccgctaccttaagagag
	oYC911_BNLF2b-DMS_fw-2N_tm61	ACACTCTTTCCCTACACGACGCTCTTCCGATCTNNatccgctaccttaagagag
	oYC912_BNLF2b-DMS_fw-3N_tm61	ACACTCTTTCCCTACACGACGCTCTTCCGATCTNNNatccgctaccttaagagag
	oYC913_BNLF2b-DMS_fw-4N_tm61	ACACTCTTTCCCTACACGACGCTCTTCCGATCTNNNNatccgctaccttaagagag
	oYC914_BNLF2b-DMS_fw-5N_tm61	ACACTCTTTCCCTACACGACGCTCTTCCGATCTNNNNNatccgctaccttaagagag
	oYC915_BNLF2b-DMS_fw-6N_tm61	ACACTCTTTCCCTACACGACGCTCTTCCGATCTNNNNNNcCAGTAGGTCCACTATGAGT
	oYC916_BNLF2b-DMS_rv-1N_tm61	TGGAGTTCAGACGTGTGCTCTTCCGATCTNGCTACCTTAGGACCGTTATAGTTA
	oYC917_BNLF2b-DMS_rv-2N_tm61	TGGAGTTCAGACGTGTGCTCTTCCGATCTNNGCTACCTTAGGACCGTTATAGTTA
	oYC918_BNLF2b-DMS_rv-3N_tm61	TGGAGTTCAGACGTGTGCTCTTCCGATCTNNNGCTACCTTAGGACCGTTATAGTTA
	oYC919_BNLF2b-DMS_rv-4N_tm61	TGGAGTTCAGACGTGTGCTCTTCCGATCTNNNNGCTACCTTAGGACCGTTATAGTTA
	oYC920_BNLF2b-DMS_rv-6N_tm61	TGGAGTTCAGACGTGTGCTCTTCCGATCTNNNNNGCTACCTTAGGACCGTTATAGTTA
	oYC921_BNLF2b-DMS_rv-6N_tm61	TGGAGTTCAGACGTGTGCTCTTCCGATCTNNNNNNGCTACCTTAGGACCGTTATAGTTA

bioRxiv preprint doi: https://doi.org/10.64898/2026.08.23.746553; this version posted August 24, 2026. The copyright holder for this preprint (which was not certified by peer review) is the author/funder, who has granted bioRxiv a license to display the preprint in perpetuity. It is made available under a CC-BY 4.0 International license.

Data S1. Design and annotation of the viral microprotein libraries.

Sheet S1a lists all 52,979 elements of the 50K viral smORF library and sheet S1b all 4,107 elements of the enriched library, with source virus, accession, genomic coordinates, start codon, annotation status, encoded amino acid sequence, subpool assignment and control class.

Data S2. Pooled 10-day growth screens with the pANDORA 50K and the Enriched library of viral smORFs.

Sheets S2a and S2b give per-element read counts, normalized abundances, $\log_2$ fold changes, Mann-Whitney P values and Benjamini-Hochberg-adjusted P values for the K562 and RPE-1 screens, respectively. Sheets S2c and S2b give the same quantities as S2a and S2b for the K562 and RPE-1 enriched-library screens.

Data S3. Pooled screens for all targeted secondary screens.

Sheet S3a, MHC-I surface expression screen in A375; sheets S3b and S3c, MHC-I screens in RPE-1 without and with interferon-γ stimulation; sheets S3d and S3e, survival screens in K562 under ER stress induced by thapsigargin and brefeldin A, respectively.

Data S4. Perturb-seq summary statistics.

Energy distance to the negative controls, energy-test FDR, number of differentially expressed genes and cluster assignment for 3,412 perturbations passing the post-QC criteria for perturb-seq processing.

Data S5. Affinity-enrichment and immunoprecipitation mass spectrometry.

Sheets S5a to S5d report $\log_2$ fold changes and Benjamini-Hochberg-adjusted P values for every bait–prey pair underlying Figs. 2, 3, 4 and 5, respectively.

Data S6. Fixed Perturb-seq custom probe set for ORF calling.

Custom probe sequences for fixed Perturb-seq with 10X Flex Gene Expression technology.
